# Multiple small bowel perforations due to cytomegalovirus related immune reconstitution inflammatory syndrome in an HIV patient: A case report

**DOI:** 10.1097/MD.0000000000026605

**Published:** 2021-07-16

**Authors:** Yanli Wang, Xuyong Lin, Yuji Li, Ying Wen

**Affiliations:** a Infectious Diseases Department, The First Affiliated Hospital of China Medical University, Shenyang, Liaoning Province, China; b Pathology Department, The First Affiliated Hospital of China Medical University, Shenyang, Liaoning Province, China; c Gastrointestinal Surgery, The First Affiliated Hospital of China Medical University, Shenyang, Liaoning Province, China.

**Keywords:** case report, cytomegalovirus infection, human immunodeficiency virus, immune reconstitution inflammatory syndrome, small bowel perforations

## Abstract

**Rationale:**

The presentation of multiple intestinal perforations is a severe complication of enteric cytomegalovirus (CMV) infection, sometimes associated with immune reconstitution inflammatory syndrome (IRIS) after the initiation of antiretroviral therapy (ART) in patients with human immunodeficiency virus (HIV). Here we reported a rare case of a patient with HIV infection who developed multiple perforations in the small bowel shortly after ART initiation without any prodromal gastrointestinal symptoms. We also reviewed the literature of reported cases to clarify their clinical characteristics for early diagnosis and rapid intervention.

**Patient concerns:**

A patient with HIV presented with fever after 16 days of ART initiation and was admitted to our hospital. He was treated with intravenous ganciclovir due to persistent CMV viremia. The fever resolved 10 days later. However, he reported persistent left lower abdominal pain.

**Diagnoses:**

The patient was diagnosed with multiple small bowel perforations, CMV-related IRIS, and acquired immune deficiency syndrome. An upright abdominal x-ray in a tertiary level hospital revealed bilateral moderate intraperitoneal free air. We performed a pathological examination and metagenomic next-generation sequencing. CMV enteritis was confirmed by immunohistochemical staining and other opportunistic infections were excluded by metagenomic next-generation sequencing.

**Interventions:**

The patient was treated with intravenous ganciclovir and 24 hours later, the patient underwent exploratory laparotomy. Partial resection and surgical repair of the small intestine were performed.

**Outcomes:**

The patient ultimately died from intestinal obstruction and septic shock 55 days after surgery.

**Lessons:**

Perforations due to CMV-related IRIS are very rare, and usually appear shortly after ART initiation. Most cases lack the prodromal symptoms of abdominal pain and diarrhea. Intestinal perforations are lethal, and early detection and surgical treatment are lifesaving.

## 1 Introduction

In patients infected with human immunodeficiency virus (HIV), cytomegalovirus (CMV) retinitis is the most common clinical manifestation of CMV end-organ disease, followed by gastrointestinal tract involvement. Although CMV infections can affect the entire gastrointestinal tract, it frequently involves the esophagus and the colon.^[1]^ Severe complications include gastrointestinal bleeding, colonic perforation, bowel obstruction, and toxic megacolon.^[2]^ Although it is very rare, bowel perforation could be the presenting feature of CMV-related immune reconstitution inflammatory syndrome (IRIS) in HIV-infected patients.^[3–6]^ This is a case report of a 28-year old man with HIV infection who presented with multiple small bowel perforations 26 days after the initiation of antiretroviral therapy (ART), without any prodromal gastrointestinal symptoms.

## 2 Case presentation

A 28-year-old man presented with several purple-blue nodules on the face and neck of one-month duration. HIV antibody testing was positive and his nadir CD4^+^ T-cell count was 25 cells/μL. After a skin lesion biopsy, the patient was diagnosed with Kaposi's sarcoma. The CMV-DNA load was 6.0 × 10^4^ copies/mL, CMV antibody (IgM) was <8 U/mL (0–18 U/mL), and CMV antibody (IgG) was 12.3 U/mL (0–12 U/mL). No abnormalities were found on funduscopic examination. The patient was found to have specific antibodies to *Treponema pallidum* and rapid plasma reagin test titer was positive with a titer of 1:2. The patient was treated with benzathine penicillin for 3 weeks. The patient was started on antiretroviral therapy (ART) with a regimen of lamivudine 300 mg daily, tenofovir disoproxil 300 mg daily, and nevirapine 200 mg twice daily.

After 16 days of ART initiation, the patient presented with fever and was admitted to our hospital. His highest recorded temperature was 38.9°C, accompanied by chills. He denied abdominal pain, diarrhea, or hematochezia. Routine blood tests showed a leukocyte count of 5.76 × 10^9^ cells/L (3.5–9.5  × 10^9^ cells/L), lymphocyte of 1.29 × 10^9^ cells/L (1.1–3.2 × 10^9^ cells/L), granulocyte of 4.04 × 10^9^ cells/L (1.8–6.3 × 10^9^ cells/L), hemoglobin (Hb) of 87 g/L (130–175 g/L), and platelet (PLT) of 348 × 10^9^ cells/L (125–350 × 10^9^ cells/L). The level of C-reactive protein was 88.7 mg/L (0–8 mg/L), and procalcitonin was 0.66 ng/mL (0–0.05 ng/mL). Toxoplasma IgG and IgM antibodies were negative. Liver and kidney function tests were within the normal range. The HIV RNA load was 2.46 × 10^3^ copies/mL and his CD4^+^ T-cell count had already increased to 75 cells/μL. The CMV-DNA load was 1.3 × 10^4^ copies/mL. The patient was treated with intravenous ganciclovir for persistent CMV viremia.

**Figure 1:**
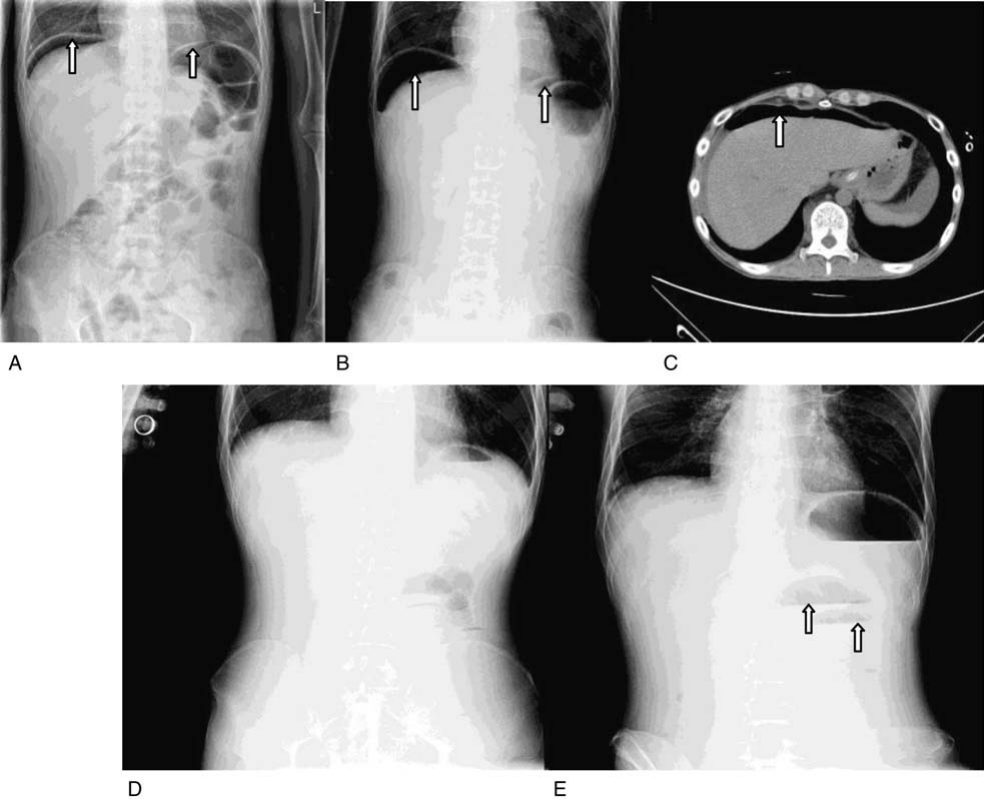
(A) An upright abdominal x-ray in a tertiary level hospital revealed bilateral moderate intraperitoneal free air. (B) An upright abdominal x-ray revealed bilateral massive intraperitoneal free air. (C) The image of abdominal CT showed free gas with a low diaphragm. (D) An upright abdominal x-ray did not show any free gas or liquid gas levels. (E) An upright abdominal x-ray showed visibly dilated intestines and gas-fluid levels.

Ten days later, the fever resolved. However, the patient reported persistent left lower abdominal pain which was spastic and tolerable. He denied abdominal distension, nausea, or vomiting. The patient was conscious and had a normal blood pressure of (BP) 115/65 mmHg, respiratory rate (R) of 16 bpm, pulse rate (P) of 88 bpm, and body temperature (T) of 36.8°C. Physical examination showed a soft and flat abdomen, left lower abdominal tenderness, without rebound pain or rigidity. Bowel sounds were 4/min. An upright abdominal x-ray in a tertiary level hospital revealed bilateral moderate intraperitoneal free air (Fig. 1A). Intestinal perforation was suspected. The patient and his family declined surgery and temporarily agreed to conservative treatment. He underwent gastrointestinal decompression and was given proton pump inhibitors, ertapenem combined with levofloxacin, and nutritional support. ART was discontinued.

**Figure 2:**
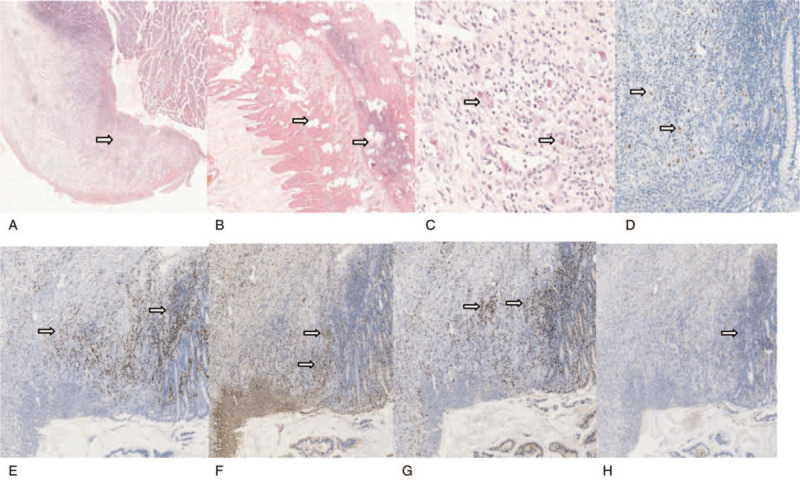
(A) Histopathological analysis showed destruction of the mucosa, submucosa, and muscular layers with neutrophilic infiltration and granulation tissue formation (×200 HE). (B) The serosa and muscular layer showed pyogenic necrosis and neutrophilic infiltration (×20 HE). C. There were intranuclear and intracytoplasmic inclusions, typical of cytomegalovirus (×200 HE). (D) Mucosa and submucosa macrophages express CMV antigens (×100 immunohistochemistry). (E) MUM1^+^plasma cells infiltration (x40 immunohistochemistry). (F) CD68^+^ tissue cells infiltration (×40 immunohistochemistry). (G) CD8^+^ lymphocytes infiltration (×40 immunohistochemistry). (H) CD4^+^ lymphocytes infiltration (×40 immunohistochemistry).

Twenty-four hours later, the vital signs were BP 110/70 mmHg, R 18 bpm, P 130 bpm, and T 38.4°C. The patient had left upper and right lower abdominal tenderness, rebound pain throughout the entire abdomen with rigidity. A repeat of the upright abdominal x-ray showed massive bilateral intraperitoneal free air (Fig. 1B) and abdominal Doppler ultrasound showed pneumoperitoneum and pelvic effusion. The abdominal computed tomography showed free gas with a low diaphragm (Fig. 1C). The repeated blood test showed a leukocyte count of 8.74 × 10^9^/L, lymphocyte of 0.65 × 10^9^/L, granulocyte of 7.96 × 10^9/^L, Hb of 103 g/L, and PLT of 338 × 10^9^/L. Alanine transaminase was 102 U/L (9–50 U/L) and creatinine was 92 μmol/L (59–104 μmol/L). An exploratory laparotomy was performed. A total of 1000 mL of suppurative peritoneal fluid was cleared. Multiple perforations at 40, 50, 65, 140, and 240 cm proximal to the terminal ileum were found at the anti-mesenteric border of the small bowel, the largest diameter of which was 1 cm. Furthermore, multiple localized discolorations on the serosal surface of the small intestine were observed indicating multiple deep ulcers (20, 80, 100, and 220 cm proximal to the terminal ileum). Partial enterectomy (35 cm) and surgical repair of the small bowel were performed. Histopathological analysis showed destruction of the mucosa, submucosa, and muscular layers with neutrophilic infiltration and granulation tissue formation (Fig. 2A). The serosa and muscular layer showed pyogenic necrosis and neutrophilic infiltration (Fig. 2B). There were intranuclear and intracytoplasmic inclusions, typical of CMV (Fig. 2C). CMV enteritis was confirmed by hematoxylin-eosin staining and immunohistochemistry (Fig. 2D). There were a variety of inflammatory cellular infiltration, including MUM1^+^ plasma cells (Fig. 2E), CD68^+^ tissue cells (Fig. 2F), CD8^+^ lymphocytes (Fig. 2G), and a small amount of CD4^+^ lymphocytes (Fig. 2H). The presence of intestinal Kaposi's sarcoma was excluded. In order to identify other possible co-infectious pathogens, formalin-fixed and paraffin-embedded (FFPE) samples from the resected bowel were sent to BGI PathoGenesis Pharmaceutical Technology (BGI-Shenzhen) for metagenomic next-generation sequencing, which indicated CMV mono-infection without co-infections of salmonella, tuberculosis, histoplasmosis, non-tuberculous mycobacteria, cryptococcosis, amebiasis, microsporidiosis, or schistosomiasis. The patient received intravenous ganciclovir 5 mg/kg twice daily for another 2 weeks followed by 5 mg/kg/day for 1 month. The CMV-DNA load was already <500 copies/mL. The patient denied any fever or abdominal pain. He was discharged 17 days after surgery and continued to take oral ART without oral ganciclovir for secondary prophylaxis.

One month after discharge, the patient was re-admitted for abdominal pain and vomiting. The patient was conscious. He had discontinued ART without consulting his physician. The vital signs were T 36.5°C bpm, 110/76 mmHg, P 103 bpm, and R 16 bpm. An upright abdominal x-ray did not show any free gas or liquid gas levels (Fig. 1D). However, 24 hours later, the patient's condition worsened with a fever and decreased blood pressure. The vital signs were BP 90/48 mmHg, P 140 bpm, and T 38.4°C. Routine blood tests showed a leukocyte count of 5.52 × 10^9^/L, lymphocyte of 0.38 × 10^9^/L, granulocyte of 4.35 × 10^9^/L, Hb of 88 g/L, and PLT of 158 × 10^9^/L. Amylase, lipase, myocardial enzymes, liver, and renal function tests were within the normal range. An upright abdominal x-ray showed visibly dilated intestines and gas-fluid levels (Fig. 1E). Intestinal obstruction due to adhesions and subsequent septic shock was diagnosed. The patient received fluid resuscitation, meropenem, dopamine, and underwent gastrointestinal decompression (Table 1). The patient's prognosis was poor and unfortunately, he died 55 days after surgery.

**Table 1:** Timeline.

	HIV-positive detection	ART for 16 days	ART for 26 days	24 h After intestinal perforation	48 Days after intestinal perforation
Diagnosis	Kaposi's sarcoma and AIDS	CMV Infection and IRIS	Intestinal perforation	Purulent peritonitis	Adhesive intestinal obstruction
CD4^+^ cell count, cells/μL	25	75	No detection	No detection	No detection
HIV RNA, copies/mL	No detection	2.46 × 10^3^	No detection	No detection	No detection
CMV DNA, copies/mL	6.0 × 10^4^	1.3 × 10^4^	No detection	No detection	<500
Symptoms	Purple blue nodules on the face and neck	Fever	Persistent left lower abdominal pain	Tenderness pain in the left upper abdomen and right lower abdomen and whole abdominal rebound pain and muscle tension.	Abdominal pain and vomiting
ART	3TC, TDF, nevirapine	3TC, TDF, nevirapine	Discontinued ART	Discontinued ART	3TC, TDF, nevirapine
Treatment	Benzathine penicillin	Ganciclovir	Gastrointestinal decompression, proton pump inhibitors, ertapenem combined with levofloxacin, and nutritional support	Partial enterectomy and surgical repair of small bowel. Intravenous ganciclovir	Meropenem, rehydration transfusion, dopamine, gastrointestinal decompression

ART = antiretroviral therapy, CMV = cytomegalovirus, IRIS = immune reconstitution inflammatory syndrome.

## 3 Discussion and conclusions

In this case report, the multiple small bowel perforations were associated with CMV-related IRIS. Although the HIV RNA load before ART initiation was unavailable, this patient met the criteria for IRIS in terms of presentation of new signs and symptoms of CMV-associated perforations in the small bowel, a low HIV RNA load, and an obvious increase in CD4^+^ T-cell count after ART initiation.

Only five CMV-associated IRIS bowel perforations, including ours, have been reported in the literature,^[1–4]^ and are summarized in Table 2. The duration of time from ART initiation to the development of bowel perforation was within 2 months and the majority of cases lacked the prodromal symptom of diarrhea. All of these cases were exclusively associated with men with same-sex sexual encounters and small bowel perforations were commonly involved. Our patient demonstrated severe secondary purulent peritonitis pre-operatively, which was the cause of death. Shortening the time from the perforation to the surgical procedure is crucial for survival; therefore, early diagnosis and treatment are necessary. Non-traumatic small bowel perforations are generally rare,^[7,8]^ and the common causes include tuberculosis, Crohn disease, and malignancies.^[9]^ The most common cause among HIV-infected individuals is CMV infection, although there has been a dramatic decrease since the development of ART. Kaposi sarcoma and lymphoma are also occasionally associated with small bowel perforations.

**Table 2:** Summary of reported cases with bowel perforation due to cytomegalovirus related immune reconstitution inflammatory syndrome in HIV-infected patients.

Case	Age	Sex	CD4 cell count, cells/μL	CMV retinitis	Duration post-ART	Manifestations and prognosis	Treatment
Gutiérrez-Delgado et al, 2017^[3]^	40	M gay	54	Y	1 mo Unmasking IRIS	No diarrhea Jejunal perforation Discharged on 17th postoperative day Readmitted 15 days later Colonic perforation Discharged without mentioning further follow-up	Oral valganciclovir Side-to-side anastomosis colostomy Intravenous ganciclovir ART was resumed Colostomy and a mucocutaneous fistula
Lee et al, 2019^[4]^	32	M	From 25 to 33	NM	53 days Unmasking IRIS	Post-ART diarrhea and CMV colitis Free air below the left-sided hemidiaphragm Jejunal perforation Survived at 30-mo follow-up	Intravenous ganciclovir Oral valganciclovir Surgical repair with peritoneal toileting ART was resumed
von Both et al, 2008^[5]^	40	M gay	164	NM	14 days Paradoxical IRIS	Diarrhea at pre-ART and acute ulcerous colitis Pneumoperitoneum Perforation Survive at 24-mo follow-up	subtotal colectomy Ganciclovir
DeRiso 2nd, et al, 1989^[6]^	40	M	From 135 to 395	NM	2 mo Unmasking IRIS	No diarrhea No air on abdominal radiograph Murky free peritoneal fluid Three jejunal perforations Discharged on 21st postoperative day without further follow-up	Partial enterectomy and enteroenterostomy repair Ganciclovir
Our patient	28	M gay	From 25 to 75	NM	26 days Unmasking IRIS	No diarrhea Five ileal perforations Discharged on 21st postoperative day Readmitted 30 days later due to bowel obstruction and severe sepsis Patient died	Partial enterectomy and enteroenterostomy repair Ganciclovir

ART = antiretroviral therapy, CMV = cytomegalovirus, F = female, IRIS = immune reconstitution inflammatory syndrome, M = male, NM = not mentioned or not done, DR = drug resistance of ART.

The most common presenting symptoms of CMV gastroenteritis are fever, abdominal pain, and diarrhea, whereas disease limited to the small bowel may be asymptomatic. CMV enteritis presenting with perforation in HIV-infected patients has been associated with high mortality due to postoperative complications including reperforation, bowel obstruction, severe sepsis, and multi-organ failure.^[10]^ CMV colitis could also be a manifestation of unmasking or paradoxical IRIS.^[11–13]^ The pathogenesis of gastrointestinal CMV disease is believed to be through submucosal vasculitis with thrombosis resulting in ischemia, ulcers, thinning of the intestinal wall, subsequent perforation, and gangrene.^[14]^ Histologic examination showed multiple areas of mucosal ulceration with acute and chronic inflammation, in addition to transmural inflammation and necrosis at the perforation sites. The criterion standard for diagnosis is the observation of cytomegalic cells with viral inclusion bodies in epithelial, endothelial, smooth muscle, and inflammatory cells.^[15,16]^ CMV infection is confirmed by immunohistochemical testing for an immediate early antigen. Real-time polymerase chain reaction and CMV culture are alternative laboratory methods. An important diagnostic tool, metagenomic next-generation sequencing using formalin-fixed and paraffin-embedded samples of lesions was significant in this case to establish differential diagnoses among various pathogens such as tuberculosis, non-tuberculous mycobacteria, histoplasmosis, and salmonella, which could all be associated with bowel perforations in patients with HIV.

In conclusion, bowel perforations shortly post-ART initiation could be considered as CMV-related IRIS in HIV-infected individuals. Preemptive anti-CMV therapy is not recommended in HIV-infected individuals with asymptomatic viremia. However, they have been proven effective in advanced cases.^[17,18]^ Routine enteroscopy and capsule endoscopy screening have not been recommended. In order to prevent life-threatening CMV colitis, early ART initiation and maintenance of high CD4^+^ T-cell counts are necessary. Being aware of atypical manifestations of the disease and rapid initiation of individualized therapy is also crucial. Last but not least, identifying high-risk individuals and using preemptive anti-CMV therapy may be lifesaving. Assessment and monitoring of patients should be done by HIV specialists and those with increased risk factors of mortality may benefit from early optimal interventions.

## Author contributions

**Data curation:** Xuyong Lin, Yuji Li.

**Formal analysis:** Xuyong Lin, Yuji Li.

**Writing – original draft:** Yanli Wang.

**Writing – review & editing:** Ying Wen.

## References

[1] Cheung TW Teich SA. Cytomegalovirus infection in patients with HIV infection. Mt Sinai J Med 1999;66:113–24. 10100416

[2] Marques O Jr Averbach M Zanoni EC Corrêa PA Paccos JL Cutait R. Cytomegaloviral colitis in HIV positive patients: endoscopic findings. Arq Gastroenterol 2007;44:315–9. 18317650 10.1590/s0004-28032007000400007

[3] Gutiérrez-Delgado EM Villanueva-Lozano H García Rojas-Acosta MJ Miranda-Maldonado IC Ramos-Jiménez J. A case report of small bowel perforation secondary to cytomegalovirus related immune reconstitution inflammatory syndrome in an AIDS patient. Ann Med Surg 2017;13:20–3. 10.1016/j.amsu.2016.11.001 PMC5176126 28018589

[4] Lee YC Chiou CC Wang JT Yang YC Tung SH Hsieh SM. Non-traumatic perforation of the jejunum in a human immunodeficiency virus-infected patient receiving combination antiretroviral therapy: A case report. Medicine (Baltimore) 2019;98:e18163. 31804330 10.1097/MD.0000000000018163 PMC6919416

[5] von Both U Laffer R Grube C Bossart W Gaspert A Günthard HF. Acute cytomegalovirus colitis presenting during primary HIV infection: an unusual case of an immune reconstitution inflammatory syndrome. Clin Infect Dis 2008;46:e38–40. 18199043 10.1086/526783

[6] DeRiso AJ 2nd Kemeny MM Torres RA Oliver JM. Multiple jejunal perforations secondary to cytomegalovirus in a patient with acquired immune deficiency syndrome. Case report and review. Dig Dis Sci 1989;34:623–9. 2539285 10.1007/BF01536342

[7] Kawate S Ohwada S Sano T. Ileal perforation caused by cytomegalovirus infection in a patient with recurrent gastric cancer: report of a case. Surg Today 2002;32:1088–90. 12541029 10.1007/s005950200220

[8] Tsai HC Lee SS Wann SR. Colon perforation with peritonitis in an acquired immunodefificiency syndrome patient due to cytomegalovirus and amoebic colitis. J Formos Med Assoc 2005;104:839–42. 16496064

[9] Freeman HJ. Spontaneous free perforation of the small intestine in adults. World J Gastroenterol 2014;20:9990–7. 25110427 10.3748/wjg.v20.i29.9990 PMC4123378

[10] Shah SK Kreiner LA Walker PA. Cytomegalovirus enteritis manifesting as recurrent bowel obstruction and jejunal perforation in patient with acquired immunodeficiency syndrome: rare report of survival and review of the literature. Surg Infect (Larchmt) 2012;13:121–4. 22439782 10.1089/sur.2010.098

[11] Yoshida S Mori N Honda M. Cytomegalovirus colitis in a patient with HIV infection shortly after initiation of antiretroviral therapy. ID Cases 2019;4:e00552. 10.1016/j.idcr.2019.e00552 PMC6515127 31193047

[12] Alukal J Asif M Mundada R Mcnamee WB. Recurrent cytomegalovirus colitis: a rare case of immune reconstitution inflammatory syndrome. BMJ Case Rep 2018;2018:bcr2017221121. 10.1136/bcr-2017-221121 PMC5787009 29301795

[13] Acosta RD Mays BC Wong RK. Electronic clinical challenges and images in GI. CMV colitis with immunereconstitution syndrome. Gastroenterology 2008;134:e1–2. 10.1053/j.gastro.2007.12.027 18242197

[14] Shiekh RA Yasmeen S Prindiville TP. Intestinal perforation and peritonitis in AIDS: case series and review of the literature. JK Pract 2004;11:248e256.

[15] Michalopoulos N Triantafillopoulou K Beretouli E Laskou S Papavramidis ST. Small bowel perforation due to CMV enteritis infection in an HIV-positive patient. BMC Res Notes 2013;4:45. 10.1186/1756-0500-6-45 PMC3568738 23379792

[16] Genta RM Bleyzer I Cate TR Tandon AK Yoffe B. In situ hybridization and immunohistochemical analysis of cytomegalovirus-associated ileal perforation. Gastroenterology 1993;104:1822–7. 8388840 10.1016/0016-5085(93)90665-y

[17] Mattioni S Pavie J Porcher R. Assessment of the efficacy and safety of pre-emptive anti-cytomegalovirus (CMV) therapy in HIV-infected patients with CMV viraemia. Int J STD AIDS 2015;26:306–12. 24845948 10.1177/0956462414536146

[18] Bigliano P Calcagno A Lucchini A. The outcome of HIV-positive late presenters according to detectable CMV DNA and anti-CMV treatment. Antivir Ther 2018;23:451–6. 29372886 10.3851/IMP3221

